# Punctiform Hemorrhages of the Brain in Gas Poisoning

**Published:** 1918

**Authors:** 


***Punctiform Hemorrhages of the Brain in Gas Poisoning.*** By Major F. W. Mott, R.A.M.C. (T.). Abs. from the *Jour. of Royal Army Medical Corps*, July, 1917, Vol. XXIX, N° 1.

Mott discusses the causes of punctiform hemorrhages of the brain that have been observed in cases of death from shell shock with burial, and in fatal cases of gas poisoning.

In 1907 the author noted the presence of such hemorrhages in cases of poisoning due to the inhalation of C O employed in the manufacture of nickel. In these cases the appearance was the same as in those of suicide by illuminating gas.

The author explains the presence of miliary hemorrhages in the white matter of the cerebrum and basal ganglia, and not elsewhere in the brain, on the following grounds:

1. Anoxemia causes the heart to beat faster, and thus to do more work with less oxygen, causing fatty degeneration.

2. Irritative and degenerative endothelial change in the cerebral capillaries, as shown by mitosis of the nuclei, and a fatty degeneration, made apparent by osmic acid staining. These changes may be due to C O in the serum, but they may be aggravated by pneumococcal toxin, which tends to increase fibrin and hence to favor thrombosis.

3. The anatomical condition of the vessels in the white matter of the cerebrum, where the arteries are terminal; each small artery having a separate capillary system, likewise the emerging veins.

4. A tendency to stasis may be brought about in these separate vascular systems by the failure of the heart as a force pump and suction pump, also by those respiratory conditions which lead to right heart dilation, and interference with the return of blood from the skull.

5. In gas cases, hemoglobine has been turned into pigment granules, and hence the hemorrhages may be accounted for by the occlusion of the arteries.

